# Cardiometabolic dysregulation and cognitive decline: potential role of depressive symptoms

**DOI:** 10.1093/eurpub/ckac129.493

**Published:** 2022-10-25

**Authors:** N Schmitz, S Deschenes, J McLeish

**Affiliations:** 1 Population-Based Medicine, Tuebingen University, Tuebingen, Germany; 2 McGill University, Psychiatry and Epidemiology, Montreal, Canada; 3 University College Dublin, Psychology, Dublin, Ireland

## Abstract

**Background:**

Previous studies have examined associations of cardiometabolic factors with depression and cognition separately.

**Aims:**

To determine if depressive symptoms mediate the association between cardiometabolic factors and cognitive decline in two community studies.

**Methods:**

Data for the analyses were drawn from the Rotterdam Study, the Netherlands (n = 2940), the Whitehall II study, UK (n = 4469) and the Canadian Longitudinal Study on Aging, Canada (n = 13,720).

**Results:**

Mediation analyses suggested a direct association between cardiometabolic factors and cognitive decline and an indirect association through depression: poorer cardiometabolic status at time 1 was associated with a higher level of depressive symptoms at time 2 (standardised regression coefficient 0.07 and 0.06, respectively), which, in turn, was associated with greater cognitive decline between time 2 and time 3 (standardised regression coefficient of -0.15 and -0.41, respectively).

**Conclusions:**

Evidence from three independent cohort studies suggest an association between cardiometabolic dysregulation and cognitive decline and that depressive symptoms tend to precede this decline.

**Key messages:**

• Cardiometabolic dysregulation and depression might increase cognitive decline.

• The association between cardiometabolic dysregulation and cognitive decline might be mediated by depression.

